# A permutation-based approach using a rank-based statistic to identify sex differences in epigenetics

**DOI:** 10.1038/s41598-023-41360-6

**Published:** 2023-09-08

**Authors:** Alice J. Sommer, Jude Okonkwo, Jonathan Monteiro, Marie-Abèle C. Bind

**Affiliations:** 1 Institute for Medical Information Processing, Biometry, and Epidemiology, Faculty of Medicine, Ludwig-Maximilians-University München, Munich, Germany; 2 Institute of Epidemiology, Helmholtz Zentrum München, Neuherberg, Germany; 3 Columbia Vagelos College of Physicians and Surgeons, New York, NY USA; 4 Department of Biostatistics, Massachusetts General Hospital, Boston, MA USA; 5 Department of Medicine, Harvard Medical School, Boston, MA USA

**Keywords:** Medical research, Epidemiology, DNA methylation

## Abstract

Epigenetic sex differences and their resulting implications for human health have been studied for about a decade. The objective of this paper is to use permutation-based inference and a new ranked-based test statistic to identify sex-based epigenetic differences in the human DNA methylome. In particular, we examine whether we could identify separations between the female and male distributions of DNA methylation across hundred of thousands CpG sites in two independent cohorts, the Swedish Adoption Twin study and the Lamarck study. Based on Fisherian p-values, we set a threshold for methylation differences “worth further scrutiny”. At this threshold, we were able to confirm previously-found CpG sites that stratify with respect to sex. These CpG sites with sex differences in DNA methylation should be further investigated for their possible contribution to various physiological and pathological functions in the human body. We followed-up our statistical analyses with a literature review in order to inform the proposed disease implications for the loci we uncovered.

## Introduction

### Sex-based epigenetic differences and phenotypic diversity

Given that most of the genome is shared between all animals, scientists have long wondered the processes underlying the phenotypic diversity in nature. In addition to pre- and post-transcription processes (e.g., gene splicing) that increase the diversity of proteins produced in animal species, epigenetic processes account for a fair amount of diversity in the gene products both within and between animal species^1,2^. There is also rising evidence that the epigenome consistently differs within a species based on sex, a phenomena worthy of investigation^3,4^. Studies have established that across the human life course, autosomal regions differentiate consistently, such that analysis of differential methylation in various genetic regions can predict biological sex accurately^5,6^. Furthermore, the literature offers a variety of methods for identifying male versus female tissue samples through differences in epigenetic markers. A recent method utilizes a sex classifier trained on whole blood samples to accurately predict both sex and instances of sex aneuploidy based on the beta values of CpG loci that differ between the samples^7^. These sex-based epigenetic differences have been potentially linked to differences in social behaviors, disease incidence, and disease outcomes. This emerging understanding has spurred studies into the impact of methylation differences in SARS-CoV-2 host cell entry genes^8^ among other relevant investigations.

### The impact of maternal stress and early developmental environments on sex-specific epigenetic differences

Although the mechanisms underlying these sex-specific epigenetic differences are not yet well known, differences in maternal care and early developmental environments have been implicated^9,10^. For example, studies have shown that stress-inducing factors, such as poor nutrition, during the gestation period can impact the offspring, both physically and psychologically, as a result of the transmission of this environmental information through the epigenome^11^. Additionally, the ways mothers treat their infants during early development has shown to influence their offspring’s social behaviors mediated by sex-specific epigenetic changes^12^. Lastly, the behavioral patterns of mothers during the reproductive and early infant development periods can be preserved and passed on epigenetically to their daughters and granddaughters^13^. Much of the work done on epigenetics has focused on the neural and psychological impact of these differences, with a large portion of these lasting modifications occurring during the embryological stages and extending into early childhood^9^. However, these epigenetic changes have proven to be incredibly dynamic through extensive changes to the epigenome over the course of a pregnancy, especially during the prenatal period^14^. Even after birth, the epigenome remains somewhat plastic and can change in response to environmental exposures, such as diet and exercise^15^.

### Molecular mechanisms underlying sex-specific epigenetic differences

Generally, much of the underlying epigenetic changes involve direct modifications to chromatin structure that can make access to gene loci more difficult for DNA transcriptase and other vital transcriptional elements. Ultimately, modifications to these histone proteins will regulate how accessible relevant DNA strands will be. Methylation and acetylation of these histone proteins are well-observed mechanisms involved in these regulatory processes^16^. In addition to epigenetic changes affecting accessibility of individual loci, we now know that entire chromosomes can be inactivated epigenitically by methylation and acetylation. Epigenetic processes in women lead to an inactivation of one X chromosome and the formation of Barr bodies, though regularly around 10–25% percent of those genes will escape. Because about 10% of the expressed micro-RNAs (mi-RNAs) in the human body is due to genetic expression originating from an X chromosome, there is a possibility that the expression of miRNA is epigenetically controlled^17^.

### Sex-based epigenetic differences and pathophysiology implications

Sex-based epigenetic differences have important implications in understanding the physiological differences between the sexes, as well as the differential pathophysiology of a variety of diseases. For example, researchers have found that a key gene, cyclin-dependent kinase 5 (*cdk5*), that is expressed in the accumbens nucleus, had higher methylation patterns in male versus female mice, which was found to correlate with increased epigenetic activation leading to longer term memory retrieval in the male versus female mice. Additionally, female mice were found to have attenuated fear memory retrieval with targeted histone acetylation of the *cdk5* promoter gene. As one can imagine, this discovery has immediate implications for research concerning medical therapy for post-traumatic stress disorder (PTSD) victims^18^.

In addition to sex-based epigenetic differences accounting for certain psychological differences, there has also been some evidence of sex-based epigenetic differences playing a role in defining neurological susceptibility to disease^19^. We already know that there are phenotypic differences in cognitive function and performance in some specific tasks between men and women that is likely due to different sex-based methylation patterns in the pre-frontal cortex^20^. Multiple sclerosis (MS), a neurological disease, has been found to be an auto-immune disease more common in women than in men. This phenomenon is suspected to be influenced by the discovery of differential epigenetic markers between men and women^21^. One study found a reduction in the percentage of differentially-regulated genes from 10% between healthy men and women to 2% percent in the diseased population, illustrating that sex-specific epigenetic dysregulation may play a role in the pathology^22^.

Prior research has also indicated that the process of acquiring the different birth sexes may also rely in part on epigenetic processes as well. In a previous study, scientists observed newborns of different sexes and found that three percent of CpG sites were differentially methylated^23^. Among these CpG sites, more than 80% of them were more methylated in the female than the male counterpart. In addition, more than 75% of sex-associated differentially methylated regions had higher methylation levels in females than in males^23^. Scientists have also found that gonadal steroids can help reduce activity of DNA methyltransferase in male embryos, which allows for the expression of masculinizing genes^24^. Their work found that female brain feminization may rely on the active suppression of these masculinization processes that occur via DNA methylation^24^. In addition, further research showed that steroid receptors present in the embryo can be targeted by transcription factors that help recruit proteins relevant to permanent epigenetic changes^25^. These hormones will then have epigenetic effects on the early nervous system that can help dictate adult differences in the brain and behavior^25^.

Sex-based epigenetic differences have been implicated in a variety of body systems and their associated pathologies. Scientists have shown that sex differences in epigenetic modifications are associated with the degree of risk factor severity in chronic obstructive pulmonary disease (COPD)^26^. Scientists have also shown that epigenetic aging rates, which are higher in men than women, are associated with a higher likelihood of cardiovascular disease^26^. Sex-based epigenetics differences also play a large role in cancer incidence and responses to treatment. It has been shown that epigenetic dysregulation can be used as a critical benchmark for likelihood of cancer initiation and adaptation^27^. The most studied sex-based differences revolve around the methylation patterns that lead to higher cancer malignancies and how mutations in DNA methyltransferase can lead to cancer promoting phenotypes in animal models^27^. Further investigations are currently underway in how differences in male and female methylation patterns can influence the ability of cancer cells to maintain diseased or non-diseased phenotypes.

### Developing a new test statistic

Here, we used a newly-developed ranked-based test statistic (i.e., the “separation statistic”) to draw permutation-based inferences about differences between sexes in the DNA methylome. We found CpG sites that had been reported by the literature to play a role in various physiological or pathological phenomena in the body. Several studies utilized standard statistical methods (e.g., linear regression) and whole human blood samples to identify hundreds of differentially-methylated CpG islands. We believe this Fisherian method will be an additional statistical tool to uncover other differentially-methylated CpG sites, allowing for improved understanding of physiological or disease processes that might prove useful in the future for continued advances in human health from an epigenetic perspective.

## Results

### Primary analysis

The primary analysis of this study compares the DNA methylation from blood samples of 362,098 CpG sites of a population of 385 Swedish Twins^28^, i.e., 385 male and 385 female adults. We use a new separation statistic whose values range from 50 to 100. The idea behind the separation statistic is to identify distributions that are far apart for two groups of interest (i.e., male and female). A 50 value indicates similar DNA methylation distributions between male and female, while a 100 value, by construction, indicates a clear separation between them. The CpG sites that we value as being “worth further scrutiny”^29,30^ have high separation statistic (i.e., $\ge$ 80) and low Fisherian p-value (i.e., $<\frac{1}{100,000}$) (see Table 1). The CpG islands presented in Table 1 show distinct distributions between males and females.

**Table 1:** Primary results.

CpG name	Scenario	Chromosome	Genomic location	Gene	Separation statistic
*cg24016844*	M>F	1	111506641	*C1orf103*	82
*cg21148594*	M>F	14	65704156	−	81
*cg04946709*	M>F	16	59789030	*LOC644649*	87
*cg11643285*	F>M	3	16411667	*RFTN1*	92
*cg04858776*	F>M	11	59318494	−	85
*cg17232883*	F>M	11	59318136	*r32542*	82
*cg03691818*	F>M	12	53085038	*KRT77*	90
*cg23719534*	F>M	15	101099284	−	85
*cg26921482*	F>M	16	2570283	*AMDHD2*	80

CpG sites that are valued to be “worth further scrutiny” with respect to sex differences after performing permutation-based inference. Scenario M>F: DNA methylation is on average higher for males than females. Scenario F>M: DNA methylation is on average higher for females than males.

**Figure 1:**
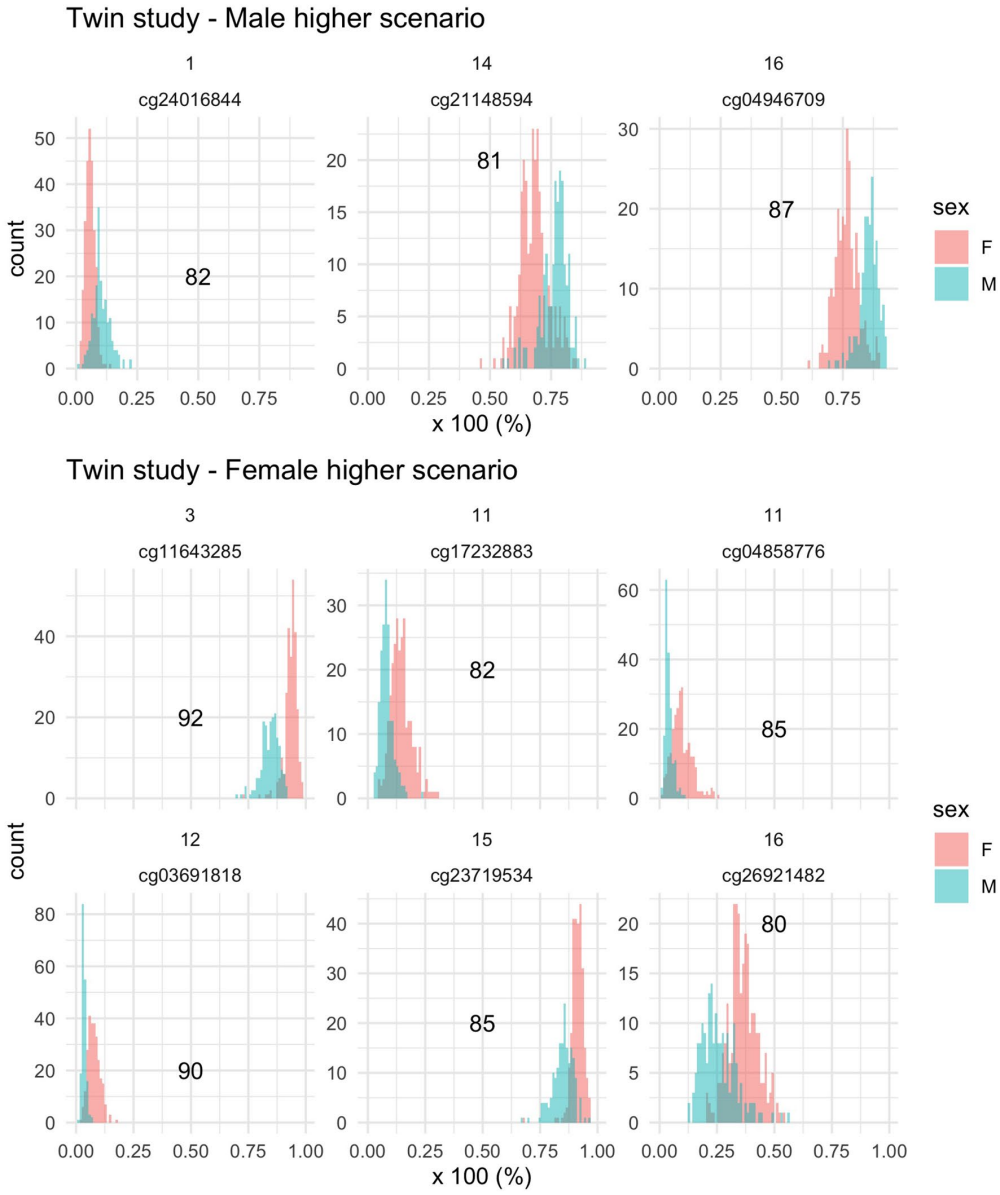
Visualization of the separation scenari in the primary analysis. Upper panel: CpG sites for which the average DNA methylation is higher in male (scenario M>F). Lower panel: CpG sites for which the average DNA methylation is higher in female (scenario F>M). Individual graph top label: Chromosome. X-axis: DNA methylation as a percentage. Number: value of the separation statistic.

To visualize and understand what the separation statistic helps depicting, we draw the DNA methylation distributions (calculated as a percentage) of the selected CpGs in Fig. 1. Even though the DNA methylation distributions among males and females do not perfectly separate, we can visually observe that for three CpGs, the male DNA methylation distribution tend to be higher than the female one (i.e., M>F), while for six CpGs, it is the opposite.

### Visual DNA methylation analysis of two pairs of heterozygote twins

**Figure 2:**
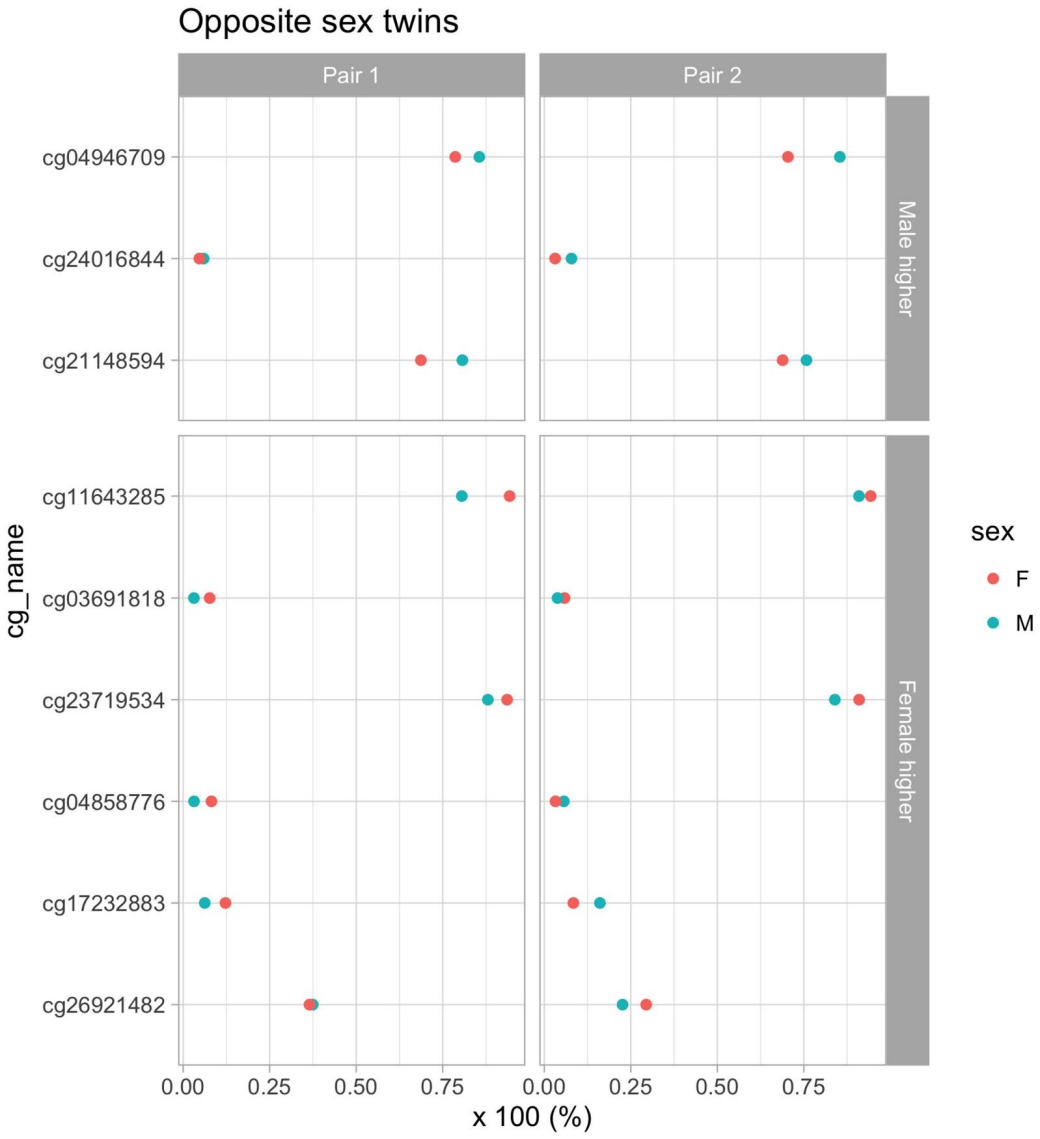
Visualization of DNA methylation separation for opposite sex twins. Upper panel: CpG sites for which the average DNA methylation is higher in males than females. Lower panel: CpG sites for which the average DNA methylation is higher in females than males. X-axis: DNA methylation as a percentage. Number: value of the separation statistic.

In the Swedish Twins Study, there are two pairs of twins that have opposite sex. For these twins, we investigate whether the CpG sites of interest (reported in Fig. 1) go in the same direction as for the overall Twin study population (see Fig. 2). Different DNA methylation separation directions are observed for *cg04858776*, *cg17232883*, and *cg26921482* for one pair of twins, but for all other sites, the direction is the same for both pairs.

### Secondary analysis

**Figure 3:**
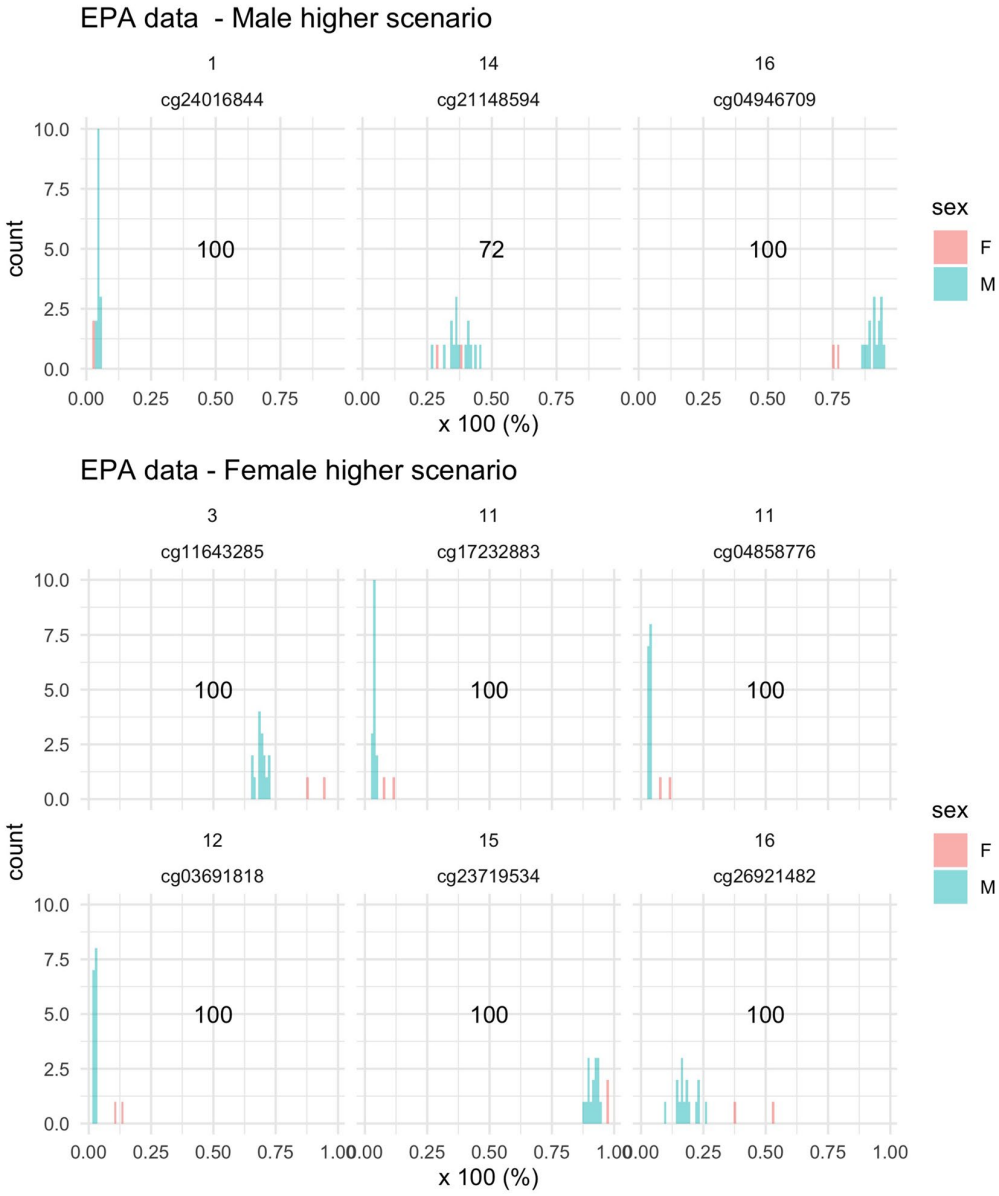
Visualization of the separation scenarios in the EPA data. Upper panel: CpG sites for which the average DNA methylation is higher for males than females. Lower panel: CpG sites for which the average DNA methylation is higher for females than males. X-axis: DNA methylation as a percentage.

To investigate whether our CpGs of interest presented similar differences in another study population, we plot the DNA methylation at these CpGs among the fifteen males and two females of the EPA study. We observe the same directions of sex differences in the EPA data (see Fig. 3). Despite the small sample size of this study (i.e., 15 males vs. 2 females), we could also visually see some separation between the two sexes. Notice that the separation statistic of *cg21148594* is lower than 80 and that a clear cut between the DNA methylation of males and females cannot be observed, which contradicts the results of our primary analysis that concluded that the sex differences of *cg21148594* were “worth further scrutiny”^29,30^. However, this result discrepancy might be due to the small sample size of our secondary analysis.

## Discussion

### Our overall approach and findings

Statistical methods have been used to uncover sex-specific methylation patterns. One study explored over 700,000 CpG sites with a linear regression relying on sex annotation, signal intensity, and comparisons of reported versus predicted parameters using whole blood samples^6^. Another study used a sex classifier to identify sex-specific methylation differences, even identifying cases of sex aneuplody, utilizing public samples even without proper annotation^7^. These studies have established the importance in the literature of validated statistical methods that are fast, reproducible, require minimal data cleaning, and robust across tissue types and annotative mishaps^6,31^. Additionally, we found that many studies utilized a linear regressions in their statistical models, necessitating the use of multi-fold data exclusion criteria, as well as statistical corrections to reach validated conclusions^6,7,31^. Here, we branched away from more traditional statistical methods and presented a new approach for uncovering these CpG sites without the required assumptions of the linear regression models found in the established literature. Instead, we used a permutation-based inference model alongside a new rank-based statistic to uncover sex-based methylation differences in human blood samples. With Fisherian p-values, we selected a threshold that allowed us to uncover CpG sites that were “worth further scrutiny”.

We were able to validate our approach by comparing our uncovered CpG sites to those uncovered by other research teams in the literature. One 2022 study found almost 400 CpG sites that were differentially methylated between females and males. The relevant CpGs were found near locations of gene regulation, e.g., enhancers^6^. Our paper uncovered similar methylation trends as visualized in Fig. 1, with a predominance of CpG sites with higher average methylation in females. This finding alongside the fact that all of our uncovered CpG sites have been found in other studies in the literature gives credence that our selected threshold value was wise, uncovering sites that have also been identified in the literature with more traditional statistical methods. Although we know that sex-based epigenetic differences may have implications for health outcomes between men and women, the pattern for those implications have not yet fully been unravelled in the literature. We now review the associated genes to CpG islands with separation statistic greater than 80 to better understand the implications of this phenomenon.

### Our biological findings contrasted to the existing literature

Chromosome 1 contains the *C1orfl03* gene, whose name is *interacting factor 1 isoform 1*, a protein-binding receptor. In contrast, the literature reveals that *LOC644649* gene is on Chromosome 16. Some researchers found that one of the alleles on this gene was implicated in schizophrenia among the Han Chinese^32^. Another gene on Chromosome 16, for which the gap statistic for sex-based differences rose above the threshold of 80, is the *AMDHD2* gene. This gene is involved in the creation of sperm and is necessary in order to break down amino sugars that sperm cells rely on^33^.

On chromosome 3 lies the *RFTN1* gene, whose gap statistic surpasses our threshold as well. This gene is necessary in order to modulate T-Cell signals, as well as allowing B-cell receptors to communicate with other immune cells^34^. As a result of the gene’s implication in the body’s autoimmunity, the gene has been further studied and found to play a role in mediating the severity of autoimmune responses, especially in inflammatory disease^35,36^. In addition, this gene locus has been implicated as a risk factor in open-angle glaucoma among teenagers and children^37^.

On Chromosome 12 lies the *KRT77* gene, which researchers often use as a convenient genetic marker in keratinocytes, and which is expressed when cellular function in the cell is normal. The dysregulation of this gene is involved in sweat gland defects and interference with normal wound healing. Researchers found that dystrophic recessive epidermolysis bullosa patients and elderly individuals share an epigenetic downregulation of this gene locus^38^.

There is a CpG site in the table that has disease implications as well. CpG site *cg04858776* is shown to be associated with a currently non-elucidated pathway that ties Alzheimer’s Disease to Type 2 diabetes^39^. In contrast, *cg17232883* (*r32542*) has been uncovered without a clear mechanism of contribution to gene expression^31^.

The CpG sites previously discussed have been uncovered in other sex-difference epigenetic studies using standard statistical methods. One study found the CpG sites: *cg24018644* (*Clorfl03*), *cg21148594*, *cg04946709*, *cg11643285* (LOC644649) and *cg23719534* to be differentially methylated between males and females, while studying the trajectory of methylation in fetal neurological development^14^. They concluded that the differential methylation were likely involved in neuropsychiatric disorders like autism spectrum disorder (ASD); disorders that are now believed to have a basis in epigenetic dysfunction during the early fetal neurodevelopmental stages^40^.

Another study analyzed these CpG sites in children a few moments after their birth to learn about the top CpG sites with sexual differentiation during an unstudied window of time^23^. The group found a 3% difference in epigenetic methylation with a 450K beadchip, including the *cg26921482* CpG site, which our gap statistic inference identified as well. Another research group found some loci that we also identified, including *cg04858776* and *KRT77*^20^, among many others as they sought to understand sex-specific methylation in the pre-frontal cortex. They understood that these sex-based differences might have implications for subtle differences in neural function or other cognitive distinctions between the sexes.

Other CpG sites reported in Table 1 include *cg21148594*, *cg04858776*, *cg17232883*, and *cg23719534*, which, to the best of our knowledge, do not have extensive literature connecting them yet to disease mechanisms. Our gap statistic implicates these CpG sites, like the others in the table, as sites that likely contribute to intriguing biomedical phenomena and are therefore promising candidates for future investigations.

### Limitations and strengths

Our study has a few limitations which could be addressed in future epigenetic studies. First, we identify an issue of sample size, illustrated by the second cohort we presented. We feel comfortable with a smaller cohort, while acknowledging a larger cohort would give us increased confidence in our ability to uncover and label relevant CpG sites as “worthy of further scrutiny”.

Additionally, our approach does not really consider the potential impact of heterogeneity in analyzed blood samples. One research team found statistical significant differences in various blood samples involved in their data set^6^. In future iterations of this project, we may consider testing our model for maintained accuracy across varying blood sample ages and processing methods that have bewildered other researchers. We might also consider testing for potentially-relevant interactions like sex-age interactions, which appear in other parts of the literature.

Our project has many strengths including the simplicity of our statistical approach. In the literature, the use of the linear regression for this work is subject to a variety of assumptions, including assumptions in regards to the underlying distribution of residuals. Our method does not rely on such assumptions, while remaining robust enough to uncover relevant CpG sites in even small sample sizes. The use of our new separation statistic could plausibly allow for faster and more nuanced discovery and/or verification of sex-specific differentially-methylated CpG sites in the future.

### Next steps

As mentioned earlier, we believe that a future avenue of investigation for these uncovered loci is an analysis of relevant sex-age interactions. Although our investigation here found that the age-related distributions potentially overlap between men and women in our study, the literature gives us reason to believe that further scrutiny of the loci in this regard could be merited.

In the broader literature, one study found after analyzing more than 400,000 loci that 7% of CpG sites studied were deferentially methylated with an association to sex, whereas 33% percent showed an association with age^41^. Additionally, the study indicated that age-sex interactions were found in CpGs that were neither hyper- or hypomethylated in DNase I hypersensitive sites in any cell type evaluated, which the authors understood to imply that this interaction was not highly heritable^41^. In the same study, the authors found that one of the published CpGs associated with lipids changes with age and sex, which ultimately implied this age-sex interaction might have relevant impact on the epigenetic regulation of metabolite loci. The research group reached a conclusion similar to the one we have posed concerning the need for further investigation into the interaction between sex and age, especially with regards to its appearance among CpG sites associated with disease loci^41^.

## Methods

### Study population

Our study population is based on 385 Swedish Twins from whom blood samples were collected up to five times. The study was originally designed to investigate the longitudinal change of methylation in association with age^28^. Phenotypes collected in this study include chronological age, sex, and zygosity.

For each participant, we used the first sample collection for analyses. For each blood sample, the DNA methylation was measured with Illumina’s Infinium 450K assay. More details about the data processing is given in^28^. A series of non-specific probes across the 450K design was identified^42^. With this in mind, we followed a strategy defined within https://github.com/sirselim/illumina450k_filtering. This strategy was used to filter probes from the Illumina methylation arrays. By the end of this process, 362,098 CpG sites remained and were tested for sex differences in DNA methylation. However, notice that this so-called sirelim method for probe filtering can now be performed more accurately with a novel method accessible on http://zwdzwd.github.io/InfiniumAnnotation.

All participants have provided written informed consents. This study was approved by the ethics committee at Karolinska Institutet with Dnr 2015/1729-31/5.

### Hypothesis testing

#### Age balance

**Figure 4:**
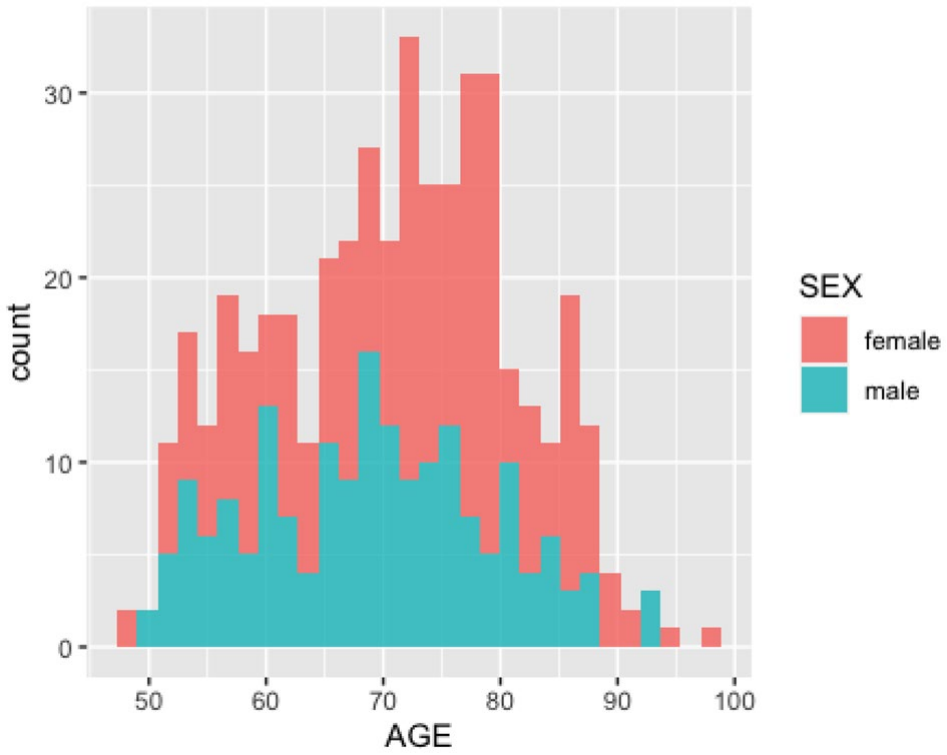
Age distribution comparison between sex.

Before testing the sharp null hypothesis of no sex differences in DNA methylation, we verify whether the participants of our data present imbalances in age distribution, because age-related changes in DNA methylation have been observed by^41^ and^28^. The age distributions among male and female fairly overlap in the data (see Fig. 4).

#### Permutation-based test of the sharp null hypothesis

We test whether DNA methylation is different among male versus female using a new test statistic. Let f(y$_{M}$) and f(y$_{F}$) denote the distributions of two mutually-exclusive male and female groups G$_M$ and G$_F$. Let Y$_{M}^{(p)}$ and Y$_{F}^{(p)}$ denote the $p^{th}$ percentiles of the distributions of G$_M$ and G$_F$, respectively. We define the separation statistic (S) as:

$$\begin{aligned} S=\left\{ \begin{array}{@{}ll@{}} Max(p)\ \text {such that}\ Y_{M}^{(p)}<Y_{F}^{(1-p)}, &\quad \text {if}\ \sum _{p=0}^{100} \mathbbm {1}(Y_M^{(p)}<Y_F^{(1-p)})\le 50 \\ Max(p)\ \text {such that}\ Y_{M}^{(1-p)}>Y_{F}^{(p)}, &\quad \text {otherwise.} \end{array}\right. \end{aligned}$$

**Figure 5:**
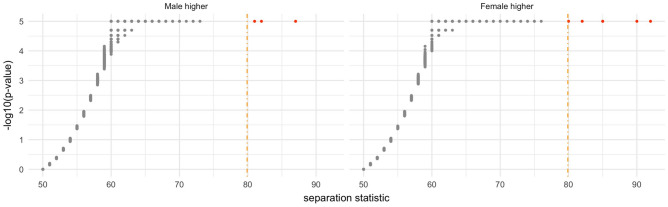
Volcano plot. Left panel: CpG sites for which the average DNA methylation is higher in male. Right panel: CpG sites for which the average DNA methylation is higher in female.

Note that by definition, the statistic ranges from 50 to 100. We chose not to rely on asymptotic arguments and instead took a Fisherian perspective (i.e., permutation-based inference)^29,43^. For each CpG sites, we tested the sharp null hypothesis of no sex-difference in DNA methylation. To approximate the “null randomization” distribution of the separation statistic, we calculated the separation statistic for 100,000 permutations of the sex labels. For each CpG, we obtain Fisherian p-value, i.e., the proportion of computed test statistics that are as large or larger than the observed test statistic. The precision for the calculated p-value in this analysis is therefore 1/1,000,000. The volcano plot on Fig. 5 reports the separation statistic of each CpG against the calculated p-value. We observe that the larger the separation statistic, the smaller the p-value.

### Secondary study

The CpG sites for which we do reject the sharp null of no sex-difference are further investigated. The data for the replication study come from a randomized epigenetic study conducted at the Environmental Protection Agency (EPA). Seventeen blinded participants were exposed for two hours, either to 0.3 ppm ozone or to clean air. The study is described by^44^. After the exposure, DNA methylation was measured at 484,531 CpG sites. We use the data only from the participants exposed to clean air.

Prior to enrollment, all participants were informed of the study procedures and potential risks, and all provided a written informed consent. The consent forms and protocol were approved by the University of North Carolina School of Medicine and the US Environmental Protection Agency. The study was registered on ClinicalTrials.gov (NCT01492517). All methods were carried out in accordance with relevant guidelines and regulations.

## Data Availability

The Swedish Twin Study dataset analysed during the current study can be inquired from the Swedish Twin Registry: https://ki.se/en/research/swedish-twin-registry-for-researchers. Details on the data processing can be found at https://github.com/sirselim/illumina450k_filtering. The US EPA data set can be requested to the corresponding author. A small portion of the data can be found at https://github.com/abele41/Human-epigenetic-study.git. All the R codes are available at https://github.com/AliceSommer/sex_epi repository.
